# Navigating Disruptive Times: How Cross-Sector Partnerships in a Development Context Built Resilience During the COVID-19 Pandemic Outbreak

**DOI:** 10.1177/00076503231169478

**Published:** 2023-05-11

**Authors:** Leona A. Henry

**Affiliations:** 1 Witten/Herdecke University, Germany

**Keywords:** COVID-19, cross-sector partnerships, CSP learning, development context, resilience

## Abstract

This article explores how cross-sector partnerships (CSPs) operating in a development context built resilience during the outbreak of the COVID-19 pandemic. Based on a qualitative analysis of eight partnerships operating in East-Africa, Central America, and Indonesia, I show how CSPs engaged in three practices of resilience building (i.e., forming unconventional alliances, mobilizing digital technologies, and building subnetworks), which allowed them to remain functional despite facing adversity. In addition to fostering their resilience, my findings show how engaging in these practices enabled the CSPs to develop new capabilities (i.e., improved abilities to engage with beneficiaries) that benefit them in the long run. Based on these insights, I advance our understanding of resilience building by unpacking this concept on a CSP level. Furthermore, by unfolding the relevance of incidental learning in a cross-sectoral setting, I widen our knowledge of learning processes in CSPs.

Cross-sector collaboration has become a popular imperative for organizations from different sectors to address collectively societal concerns they cannot tackle by themselves (Clarke & Crane, 2018; Selsky & Parker, 2005). In this article, I focus on cross-sector partnerships (CSPs) in the development context, in which actors from the private, public, and/or civil society sectors collaborate to foster local communities’ economic infrastructure (Stadtler, 2016). Such CSPs organize various activities that revolve around strengthening communities’ financial autonomy, including the provision of training, building connections to relevant markets, and establishing financial savings schemes. Due to their collaborative nature and strong reliance on face-to-face interactions, the outbreak of the COVID-19 pandemic jeopardized these CSPs’ capacity to maintain functional, raising the question how they engaged in resilience building (Williams et al., 2017; Williams & Shepherd, 2016) to sustain their activities amid this unexpected upheaval.

Existing research on resilience provides some insights into this question. For example, studies have shown how organizations can build resilience by mobilizing slack resources (Gittell et al., 2006), by developing strategic sustainability practices (Desjardine et al., 2019), or by adapting their business model (Buliga et al., 2016). Yet, existing studies offer less insight into how multi-stakeholder forms of organizing, such as CSPs, manage to remain functional when confronted with unexpected situations. In addition, while it has been recognized that organizational actors may flourish and grow during times of adversity (Hillmann & Guenther, 2021; Sutcliffe & Vogus, 2003), little is known about the mechanisms that allow them to do so. By assessing empirically how CSPs remained functional and developed new skills during the outbreak of the global COVID-19 pandemic, this article seeks to address the above-mentioned shortcomings in the literature. Hence, this article asks: How do CSPs build resilience and develop new capabilities during periods of adversity?

I explored this question in a qualitative study comprising managers and coordinators of eight CSPs supporting coffee and tea farmers located in East-Africa, Central America, and Indonesia with their agricultural production. The outbreak of the pandemic affected the CSPs’ ability to remain functional because access restrictions and social distancing ensured that they were no longer able to actively engage with farmers. In addition, the pandemic reinforced farmers’ financial instability as well as social problems, which challenged the CSPs’ ability to operate even more. My analysis reveals three practices of resilience building that the CSPs engaged in to remain functional despite this disruptive situation: (1) forming unconventional alliances, (2) mobilizing digital technologies, and (3) building subnetworks. Moreover, my findings show how the CSPs were able to develop new capabilities during the pandemic by detecting and seizing new opportunities for interacting with their beneficiaries.

The findings of this study offer three theoretical contributions: first, they contribute to the literature on resilience (Hillmann & Guenther, 2021; Linnenluecke, 2017; Raetze et al., 2021) by deepening our understanding of this construct on a CSP level. Thus, this study responds to a call for empirical insights on the mechanisms behind multi-stakeholder resilience organizing (Van der Vegt et al., 2015). Second, by unfolding how CSPs develop new capabilities during the pandemic, the findings of this article shape our understanding of the factors that allow organizations to flourish despite adversity (Christianson et al., 2009). Third, this study addresses the debate on CSP learning (Dentoni et al., 2021; MacDonald et al., 2022; Ryan & O’Malley, 2016) by showing how actors in CSPs can learn collectively from re-organizing during an unexpected event. While scholars have often emphasized learning as an important outcome of CSP collaboration, our understanding of learning processes on a partnership level remains limited. The findings of this study provide more insights into this process by highlighting the value of incidental learning (Marsick et al., 2008) in a CSP context. Finally, this study adds to the stream of research on COVID-19 organizing (Bansal et al., 2021; Muzio & Doh, 2021) by highlighting the implications of the pandemic for cross-sector collaboration. While such implications have been assessed for several areas including global value chains (Kano & Oh, 2020), corporate social responsibility (Carroll, 2021), and organizational strategy (Foss, 2020), research on COVID-19 organizing within CSPs is scarce.

The remainder of this article is organized as follows: the following section reviews prior research on resilience organizing and CSPs. The subsequent section on methods discusses my research setting, my approach to data collection, and the data analysis. In the findings section, I present the practices that allowed the CSPs studied to build resilience and the unintentional learning benefits that emerged as a result. In the discussion section, I elaborate my theoretical contribution, framing these findings in the light of resilience and CSP learning. Finally, the conclusion outlines directions for future research.

## CSPs in a Development Context and Resilience

In CSPs, actors from different sectors jointly develop solutions to address complex societal concerns (Clarke & Crane, 2018; Selsky & Parker, 2005). Prior research has shown how CSPs address such challenges through cross-sectoral learning (Stadtler & Van Wassenhove, 2016), by creating dialogue across organizational boundaries (Austin & Seitanidi, 2012) and by developing organizational capabilities (Dentoni et al., 2016). Due to their ability to pool resources and expertise across sectors, CSPs have become an important form of collective organizing for a variety of grand challenges, including education (Stadtler, 2018), peace development (Kolk & Lenfant, 2015), or climate change (Henry et al., 2022). In this article, I focus on CSPs operating in a development context, which are considered promising governance instruments to foster sustainable development (Bäckstrand, 2006; Kolk et al., 2008). The overall aim of CSPs in a development context is to improve local communities’ economic viability, which they do through the provision of different services to those communities (Beisheim et al., 2014). These include strengthening communities’ connections to relevant institutional actors (Trujillo, 2018), providing new employment opportunities (Vestergaard et al., 2020) or offering technical assistance and training (Merino & de los Ríos Carmenado, 2012). CSPs in the development context thus represent an operational form of cross-sectoral organizing that relies on frequent interaction and collaboration with beneficiaries (Stadtler, 2016).

Resulting from their operational and interactive nature, the outbreak of the global pandemic severely impacted these CSPs’ ability to remain functional and required them to build resilience. Resilience is a concept that has been widely discussed in the management literature and one that is characterized by a variety of definitions (see Linnenluecke, 2017 for an overview). In this article, I draw on the definition of resilience as “the process by which an actor (i.e., individual, organization, or community) builds and uses its capability endowments to interact with the environment in a way that positively adjusts and maintains functioning prior to, during, and following adversity” (Williams et al., 2017, p. 742). The outcome of this process is actors’ ability to remain functional despite adversity (Nava, 2022; Sutcliffe & Vogus, 2003). The management literature has shown a variety of practices that enable organizations to achieve this: for example, Gittell et al. (2006) highlight the importance of designing business models with abundant financial reserves to overcome turbulent times. Exploring airlines’ responses to 9/11, the authors put forward the importance of mobilizing slack resources to retain employees and foster commitment in times of crisis. In the context of the global financial crisis, Desjardine et al. (2019) show how strategic sustainability practices, which create interdependence and a long-term perspective, enable organizations to adapt to unexpected disturbances. Other studies have highlighted innovation (Hamel & Valikangas, 2003), experimentation, and learning (Do et al., 2022), as well as developing employee strength (Luthans, 2002) as means for organizations to remain functional during turbulent times.

While extant research on resilience thus already provides us with valuable insights, it also reveals a few shortcomings that limit constructive theorizing. To begin with, existing studies on resilience are limited with regard to how this can be achieved in cross-sectoral collaborative settings (Linnenluecke, 2017; Van der Vegt et al., 2015). Even though scholars have addressed resilience on an inter-organizational level, it has been studied primarily from the perspective of high-reliability organizing. Studies in this domain have highlighted how interorganizational collaborations are able to face adversity by quickly changing their governance modes (Berthod et al., 2017), by developing agile and trust-based coordination mechanisms (Kapucu et al., 2010), or by setting up effective boundary-spanning networks (Kapucu, 2006). While fruitful, these findings are only partly informative with regard to how CSPs, which are composed of heterogeneous and often globally dispersed stakeholders, remain functional when confronted with adversity.

Second, while extant studies frequently emphasize that times of adversity allow organizations to develop new capabilities (Lampel et al., 2009; Sutcliffe & Vogus, 2003), only few studies provide empirical evidence of this process. For one, Christianson et al. (2009) show how, following a roof collapse due to a natural disaster, the Baltimore & Ohio Railroad Museum Roundhouse redefined its identity and was able to attract more visitors than before. In a similar vein, Salvato and colleagues (2020) show how firms were able to turn strengthened relationships with community members into business opportunities after a natural disaster. Yet, beyond these insights, we still know little about the factors that allow organizational actors to acquire new skills during unexpected disruptions. Against the backdrop of the global COVID-19 pandemic, this study seeks to address the above-mentioned shortcomings in the literature and explores how CSPs remain functional in the face of adversity, as well as how they learn to develop new capabilities through this experience. As CSPs are seen increasingly as important vehicles for sustainable development (Vestergaard et al., 2020) and systemic change (Clarke & Crane, 2018), broadening our understanding of this question has both theoretical and practical implications.

## Empirical Approach and Methods

### Research Setting

To understand how CSPs built resilience and developed new capabilities during COVID-19, my study comprises a qualitative research design. The setting for this research consists of eight CSPs that aim to strengthen the economic infrastructure of local farmer communities by improving their agricultural production. The choice of CSP for this study is based on the following criteria: (a) their function lies in capacity building and implementation (Andonova et al., 2009), which implies they operate through the provision of resources (e.g., financial resources, expertise, or technology) and actively engage with their beneficiaries in this process, (b) they operate in a development context, and (c) the CSPs’ beneficiaries all produce the same agricultural commodity, in this case, coffee or tea. Hence, the CSPs were selected following a logic that Yin would refer to as “literal replication” i.e., they are expected to produce similar results (p. 103). The CSPs’ beneficiaries are coffee and tea farmer communities located in developing regions in East-Africa, Asia, and Central America. These farmer communities, which consist mainly of farmers and their families, face challenges in terms of accessing financial markets, modernizing their production processes, and stabilizing their income. While they maintain close relationships with the CSPs, the farmer communities are not considered to be part of the partnerships but are seen as allies. The CSPs under study were all founded by a focal group of organizations consisting of a combination of companies, development agencies, private foundations, or governmental organizations that have a shared interest in addressing development challenges. In line with their focus on agriculture, most of the CSPs’ partners have expertise in coffee or tea production or (sustainable) agriculture.

Within the different regions they operate in, the focal CSP members assemble additional partners such as local governments or nongovernmental organizations (NGOs), whose expertise is required to support communities successfully. Together with these local partners, the focal CSP develops, implements, and monitors so-called “intervention programs” aiming to strengthen farmers’ agricultural production. Within the intervention programs, the CSPs organize various services to support farmers’ economic development: the first and most important service provided by the CSPs constitutes a set of interactive agriculture trainings, in which the CSPs practice the use of different pesticides, show farmers how to diversify crops and sensitize them to the effects of climate change on their crop production. Second, the CSPs organize cooperatives that help farmers to improve their market access and enable them to connect to important institutions. In a similar vein, the CSPs assist farmers and their families to set up saving schemes to help stabilize their financial situation. Finally, the CSPs organize “youth clubs,” in which they provide groups of young farmers with agriculture and leadership training. During the development of these activities, farmers and their families are actively involved, to ensure these programs address their needs. Following others in the field, I thus understand the beneficiaries as recipients of the CSPs’ services, but as playing a key role in shaping their content and impact (Trujillo, 2018).

To coordinate the intervention programs, the CSPs involved in this study work with project coordinators, who oversee the general program and liaise between the different partners involved. These coordinators are either located in the regions themselves or in Europe, but regardless of their location, they keep in close contact with the staff and the farmers in the regions in which the interventions are implemented. In the regions themselves, the CSPs employ both country and local managers. Country managers are responsible for the coordination and implementation of the programs within one country, whereas local managers are responsible for the implementation of programs within one community of farmers. Both these manager types are in frequent contact with one another, the project coordinators, and with the farmer communities they work with. As all the above-mentioned actors collaborate closely with the CSPs’ beneficiaries, they were able to provide a good overview of the impact of the pandemic outbreak as well as the CSPs’ efforts at resilience building during COVID-19. A full overview of the CSPs involved in this study can be found in Table 1. To ensure anonymity, I identify the CSPs by their core capacity building focus, “Agri,” along with the first letter of the region they operate in (e.g., “AgriU” for the agriculture CSP operating in Uganda).

**Table 1.** Overview of CSPs Involved in this Study.

CSP	Focal CSP members	Beneficiaries
AgriE	Private foundation (3), development agency (1), NGO (1)	Coffee smallholder families in Ethiopia
AgriH	Private foundation (1), companies (5), development agency (1)	Coffee smallholder families in Honduras
AgriI	Companies (2), development agency (1), private foundation (1), NGO (1)	Coffee smallholder families in Indonesia
AgriK	Private foundation (1), NGO (1), companies (8)	Tea smallholder families in Kenya
AgriM	Developmental agency (1), NGO (1), companies (8)	Tea smallholder families in Malawi
AgriP	Research institutes (2), private foundation (1), development agencies (3)	Coffee smallholder families in Peru
AgriT	Private foundation (1), NGO (1), companies (8), development agency (1)	Coffee smallholder families in Tanzania
AgriU	Private foundations (3), governmental organization (1)	Coffee smallholder families in Uganda

*Note.* NGO = Nongovernmental Organization.

### Data Sources

#### Semi-structured Interviews

In terms of data sources, this study draws on semistructured interviews, informal interviews, and documentary evidence allowing for converging lines of inquiry and data triangulation (Yin, 2018). As connections to (transitional) CSPs are notoriously challenging to arrange, I collaborated with a U.K.-based partnership consultancy as well as a German Foundation involved in development assistance, which helped me to establish connections to the CSPs involved in this study. As I wanted to understand how CSPs in a development context experienced and coped with the pandemic, I was provided with contact details to regional CSP managers in developing countries that were severely impacted by COVID-19. These regional managers in turn helped me to establish contact with actors within the CSPs operating in their respective areas. As outlined in the case description, each of the CSPs in this study works with project coordinators, country managers and local managers, of which I sampled as many as possible. In total, I conducted 17 semistructured interviews with CSP actors on a variety of these three levels. The interviews were broadly organized around the CSPs’ mode of engagement with their beneficiaries, the impact of COVID-19 on community life, and how they responded during the pandemic. While I used these broader themes as a backbone to structure my interviews, I always adjusted the themes to the role of the interviewee in question and allowed them to talk about their own experiences. The interviews took place via Zoom and lasted between 45 and 60 minutes. Except for one interview during which technical issues occurred, all the interviews were audio-recorded and transcribed.

Approximately 6 months after the first round of data collection, I contacted the interviewees a second time to find out how the situation had evolved. In this second round of data collection, which was more informal, I sent out emails to all the interviewees asking them to describe to me how they were currently experiencing the pandemic in their respective countries and what activities they were engaged in. In addition, I met some of my interviewees again via Zoom in December 2021 to discuss the afore-mentioned aspects. This second round of inquiry gave me a good impression of how the pandemic situation and CSPs’ organizing efforts had developed.

#### Secondary Data

For each CSP, I analyzed a variety of secondary sources such as blog posts, press releases, and publications developed by the CSPs around COVID-19. These documents provided helpful data sources to understand the challenges the CSPs were facing during the pandemic and how they responded to them. I also used other documents, which were not related to COVID-19, such as the CSPs’ general project websites or blog posts. These helped me to develop a more thorough understanding of the CSPs involved in this study and the general organization context. Wherever I cite directly from these secondary sources in the findings section, I have slightly altered the original phrase to guarantee the anonymity of the CSPs involved. However, these secondary data sources can be provided upon request. All secondary data were also coded in ATLAS.ti.

I also took several measures to capture the beneficiaries’ perspective through secondary data: initially, I analyzed all the material that the CSPs had collected among farmers that they were willing to share with me. For example, in the case of one CSP (AgriE), I was granted access to a confidential survey sent out by the CSP to its community members, in which they reported their perceived challenges and experiences during the pandemic. For AgriU, I was given access to a confidential recording, in which the CSP discussed feedback and general impressions during COVID-19 that they had received from farmers. Finally, for AgriT, I took part in a webinar hosted by the CSP, which also involved farmers who shared their experiences regarding COVID-19. Hence, I tried to substitute for the missing interviews with the CSPs’ beneficiaries with other data sources as far as possible. Toward the end of my data collection, I noticed that no more new ideas and themes were being added to the data set, implying that I had reached thematic saturation (Guest et al., 2006). A full overview of the data sources used for this study is provided in Table 2.

**Table 2.** Overview of Data Sources.

CSP	Interviews	Secondary data sources
AgriE	Project coordinator (1), country manager (1)	Project website (1), blog post (1)
AgriH	Project coordinator (1), country manager (1)	Project website (1), general blog post (1), COVID-19-related blog posts (2), confidential results survey among beneficiaries on COVID-19 (1),
AgriI	Country manager (1)	Project website (1), blog post (1)
AgriK	Project coordinator (1), country manager (1)	Project website, general blog posts (2)
AgriM	Project coordinator (1), country manager (1)	Project website, general blog post (1)
AgriP	Project coordinator (1), country manager (1)	Project website (1), general blog post (1)
AgriT	Project coordinator (1), country manager (1)	Project website (1), webinar (1), general blog posts (3)
AgriU	Project coordinator (1), country manager (1), local managers (2)	Project website (1), confidential online CSP meeting (1), general blog post (1), COVID-19 related blog posts (2), press release (1)
*#*	17	25
Other data sources	Informal interviews The Partnering Initiative (7)	

### Data Analysis

To understand how the CSPs built resilience and developed new skills in the aftermath of the COVID-19 pandemic, I relied on an open-ended theory-building approach, which started during the data collection stage and involved several iterative cycles (Strauss & Corbin, 1998). To analyze my data systematically, I collected all data sources in an ATLAS.ti database. My further analytical process involved coding, categorizing, and abstracting to higher-level concepts (Gioia et al., 2013). I present the main three steps of this process below.

In the first step, I coded all data passages that were informative in terms of understanding the influence of the pandemic on CSPs and their work with farmer communities. I coded instances in the data in which CSP members mentioned how the outbreak affected their ability to sustain their activities and the lives of farmer communities. In this first round of coding, I also focused on data passages in which informants talked about how they had responded to the outbreak of the pandemic (i.e., what they “did about it”). Finally, I coded passages that were informative in terms of changes in CSP overall organizing patterns triggered by these responses. From these passages, I created a set of first-order codes that were close to the raw data and mostly in vivo.

In a second round of coding, I aggregated the first-order codes created in the first step into second-order themes, which allowed for a more theoretical interpretation of those codes. During this step, I iterated back and forth continually between the literature and my data to understand what the challenges and responses I had observed in step 1 were actually “a case of.” While I did not settle on a final theoretical interpretation of my data at this point, this process did enable me to understand that the CSPs were affected by the pandemic, as they were unable to continue their activities. On top of that, the pandemic caused severe financial and social problems for farmer communities, which further challenged the CSPs’ ability to sustain their work. Second, I came to understand that the practices the CSPs developed to build resilience could be categorized in different ways, based on their different underlying mechanisms. For example, I framed the practice implying that the CSPs sought support from atypical actors, as “forming unconventional alliances.” In a similar vein, I labeled the CSPs’ efforts to deploy digital platforms and technologies as “mobilizing digital technologies” and the practice of restructuring communities into smaller networks and groups as “building subnetworks.” In this step of coding, I also identified and grouped a set of first-order codes that were informative in terms of the unintended consequences these responses had on the CSPs’ overall organizing efforts. An insight that I derived here was that the CSPs developed new capabilities, which pertained to (a) the development of innovative training formats and (b) the development of new communication channels.

**Figure 1.**
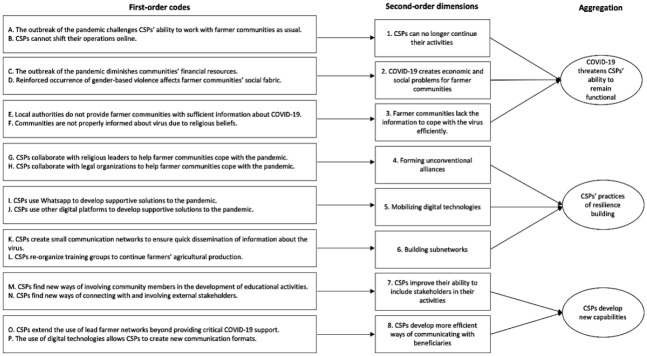
Data Structure.

In the final step of coding, I thickened my analysis and provided a theoretical interpretation of my data by building a set of aggregate dimensions. Drawing on the literature on resilience building (Sutcliffe & Vogus, 2003; Williams et al., 2017), I labeled the CSPs’ challenges to sustain their activities during the outbreak of the pandemic as “COVID-19 outbreak threatens CSPs’ ability to remain functional” and the practices they developed during this period as “CSPs’ practices of resilience building.” Finally, I labeled the improvements they made to their interaction with their beneficiaries and stakeholders as “CSPs develop new capabilities.” The data structure that emerged from this process is shown in Figure 1. The analysis of secondary data sources coincided with the analysis of the interviews and followed a similar three-step approach.

## Findings

### COVID-19 Outbreak Threatens CSPs’ Ability to Remain Functional

The outbreak of the global pandemic severely impacted the ability of CSPs to remain functional (i.e., to sustain their support for their beneficiaries’ agricultural production) and continue other community activities. A variety of factors led to this situation: first and foremost, access restrictions and social distancing ensured that CSPs were no longer able to enter the farmer communities, which severely challenged their ability to continue working as usual. As the country manager in Malawi explained:

The first weeks were difficult because we could no longer engage. When you are engaging, you have life, like a class that you train to work together, or we have demonstration plots. So, a lot of our learning is learning by doing, learning in groups, learning from each other, learning through animation and all of that, which in that case is completely lost. And you are hardly seeing the people, you are not getting the responses, you are not seeing in real time what impact your messaging has had. (Country manager AgriM)

A second factor that challenged the CSPs in the continuation of their activities was the fact that the pandemic had reinforced some deep-rooted community problems, starting with financial issues. Lockdowns and access restrictions implied that farmers were no longer able to go out in the field and continue their production in the same way as before. In addition, the outbreak of the pandemic had made access to critical inputs like fertilizer more difficult (Opitz, 2020b). Besides challenges surrounding production, the CSP managers perceived that the outbreak of the virus also ensured that farmers were having difficulties selling their products. One of them explained:

It became a big challenge for the farmers, who depend on their crops and sell their crops to earn their incomes. They may have a few banana boxes to bring to the market, but you know, people are not coming to the market because markets are restricted. In the villages we don’t have supermarkets. So, their income decreased quite a lot because they could not sell much. (Country manager AgriT)

Besides financial issues, the outbreak of the pandemic caused social problems for the farmer communities, of which the most significant was that of gender-based violence. While this was not an entirely new problem, lockdowns and access restrictions reinforced its occurrence during the outbreak of the pandemic. This became problematic for the CSPs’ ability to sustain their work, as they perceived that young females were scared and were consequently no longer willing to participate in community activities or fieldwork, even after lockdowns had ended. The country manager for Malawi described the situation as follows:

What we saw was that there was a lot of teenage pregnancy and gender- based violence during that period, and we felt there was an increased need for psychosocial support. This is something we had never thought about as a partnership. (Country manager AgriM)

A final important factor that challenged the CSPs in the continuation of their activities was the fact that, according to CSP actors, farmers and their families were not properly informed about the virus and how they could protect themselves. This was either because their rural location ensured that they would not receive any governmental information or because the government spread unreliable information. For example, the country manager in Tanzania explained

After two months the government removed all the restrictions and they diminished to control the virus, so they said, “the virus no longer lives in Tanzania, we are free of the virus.” So, they also restricted importation of masks from outside the country, and they were telling people it was not necessary to wear a mask. (Country manager AgriT)

Besides misinformation spread by the authorities, the CSP managers also suspected that religious beliefs ensured that farmers and their families were not properly informed about the virus. For example, the country manager for the Ethiopian CSP described the situation as follows: “The general perception in Ethiopia is that ‘God protects us, and we are immune.’” As both the lack of governmental information as well as prevailing religious beliefs led to the fact that farmers would not protect themselves and thus caused infection rates to increase, they impeded the continuation of the CSPs’ work.

Overall, various factors thus ensured that the CSPs’ ability to continue their activities during the pandemic was threatened and so a context emerged that required resilience building efforts. In the following, I will describe in detail the practices the CSPs developed to remain functional during this situation (i.e., to build resilience).

### CSPs’ Practices of Resilience Building

#### Forming Unconventional Alliances

A first way for the CSPs to remain functional during the outbreak of the virus was by teaming up with community members or organizations they would not usually seek support from. As mentioned, a big challenge for the CSPs was the fact that in many farmer communities a strong religious belief prevailed, which led to farmers questioning the existence of the virus. To respond to this challenge and ensure farmers and their families would protect themselves nevertheless, the CSPs started to team up with religious leaders. Below, the Ethiopian CSP manager explains how he is carefully mobilizing priests to help him inform their beneficiaries about the virus:

We have to be very careful here, if we just approached them [priests] separately, it might become a very challenging conversation. So, we have the conversation about COVID-19 and how to protect ourselves with communities in their presence. We just relate the conversation in a way that the priests reinforce our arguments. So that it doesn’t look like religious pressure, but whatever we do, we just say, “do you agree with us.” Something like that, so it doesn’t look artificial. We try to make it organic. A priest has no choice but to support our argument because we also support their faith. And we emphasize that, yes, we strongly believe that God is the one who protects us, but sometimes it does not hurt to protect yourself as well. (Country manager AgriE)

In Kenya as well, the CSP started to collaborate with religious leaders to ensure farmers would take information about the pandemic seriously: “We relied a lot on them [religious leaders], because in those rural communities, the religious leaders have the most credibility. When they say ‘wash your hands,’ people will actually do it” (Country manager AgriK).

As mentioned, another severe challenge that impeded the CSPs’ functioning was the fact that the outbreak of the pandemic had reinforced the problem of gender-based violence, especially in the African countries. This was problematic for the CSPs and their work, as it ensured that young females were no longer taking part in activities such as educational programs or community meetings, even once the lockdowns had become less strict. Below, the country manager in Malawi describes how the CSP mobilized a set of new partners to respond to this pressing issue:

What we did was that we partnered with emotional support organizations and legal organizations. And as we could not go into the community as much, we sent out a radio broadcast in which we gave the telephone numbers to reach this particular lawyer. Of course, we did not call them a lawyer, because people might get scared then. But we made it very clear that “if something happens to you, you can dial this number for free and somebody will help.” (Country manager AgriM)

Thus, as shown by the findings above, a first way for the CSPs to build resilience during the pandemic consisted of forming partnerships with actors such as priests or lawyers. While these were actors that the CSPs would not usually collaborate with, they proved to be vital in the CSPs’ efforts at resilience building during this period.

#### Mobilizing Digital Technologies

A second way for the CSPs to remain functional during the pandemic was by mobilizing digital technologies and tools and turning them into collaborative solutions. While the CSPs had occasionally used digital technologies before, such as mobile phones or digital platforms, they were not part of their general activities, which changed during COVID-19. First, the CSPs started to use Whatsapp in a different manner than they had thus far. In Uganda for example, the CSP used the digital platform to develop a professionalized, daily radio broadcast with community members, which was intended to distribute information about COVID-19 and to help farmers sustain their agricultural production. As one of the local managers explains:

During the pandemic we created “Agrihour,” a daily radio show, for which we would have our local managers and field officers record the most important information about COVID-19, but also about sanitation, clean water and agriculture work on Whatsapp. So, it would be a bundled set of messages for our communities. We sent these messages to the local radio station, which would put them together and broadcast them on our behalf every day at the same time, so it really became a professional broadcast that communities could rely on. And at the end of the show, we’d make sure the listeners could call in to ask us questions and provide us with feedback for the next broadcast. (Local manager AgriU)

Besides using Whatsapp for the creation of a radio show, the CSPs started to use this technology to keep farmers’ production going as much as possible. For example, the CSP and farmers in Uganda developed a new way to communicate about agricultural practices. As one of the local managers explains:

We obviously couldn’t go into the field, so we made sure that our youth clubs [groups of young farmers] were organized in a Whatsapp group. Representatives of the groups would send our trainers pictures of the farmers, showing what they were doing on the fields. And the young farmers would get feedback on that, also via Whatsapp, or they could call in. We called this “phone farming.” (Local Manager AgriU)

In addition to increasing the use of Whatsapp, the CSPs started to use other digital platforms to cope with the economic burden caused by the situation. For example, in Tanzania, the CSP started to use a platform called “we farm” to ensure farmers would be able to sell sufficient products and generate an income despite the low coffee prices during the pandemic. As the country manager explains:

One thing we discovered during the pandemic was a mobile tool that connects farmers to potential buyers. It is a mobile connection platform that does not require the internet. So, a farmer would be able to say, “okay, I have 10 eggs, who can I sell them to?” And the platform would then help them find those buyers. Of course, it is not the same as selling coffee, but it enabled communities to at least sell other products. (Country manager AgriT)

In sum, a second way for the CSPs to build resilience during the pandemic was by mobilizing digital platforms and technologies, which enabled the CSPs to inform farmer communities about the virus, but also to sustain and diversify their agricultural production.

#### Building Subnetworks

Finally, the CSPs also restructured farmer communities into smaller networks to continue their support throughout the pandemic. Especially in regions with scarce internet connectivity, this practice of creating smaller units proved to be an efficient way to ensure the rapid diffusion of information about the virus throughout farmer communities. Doing so was challenging for the CSPs during the pandemic, as the communities often consisted of many dispersed households that they could not visit individually. On top of that, social distancing requirements made it impossible to organize large gatherings with farmers. To address this issue, the CSP in Kenya created a small network of farmers that enabled the rapid diffusion of COVID-19-related messages throughout communities. As the country manager says:

We have established a network of what we call “lead farmers,” that is, farmers who act as a representative for other farmers in their community. During the pandemic you can’t reach communities of 100 people, so you break them into groups and then you have leaders within those groups. During the pandemic we have used this system to contact those leaders and give them key messages using the phone. So that they could also be in contact with various members under them, but it reduces the number of contacts at each point. (Country manager AgriK)

Besides effective health information provision about the virus and protective measures, the CSPs used the practice of forming small networks within farmer communities to reorganize their training formats in such a way that agricultural support could be continued. Below, the country manager in Tanzania describes how the CSP created small-scale training sessions for farmers:

So, what we did is that we divided the community into groups of 5 farmers, who would come to the field in shifts. We would have a trainer there, who would bring a phone with him and connect us with the farmers. We could not really show them anything new, but we could discuss their progress, what challenges they faced, and discuss any other questions they might have. (Country manager AgriT)

Thus, a final means for the CSPs to remain functional during the outbreak of the global pandemic was by dividing farmer communities into smaller networks to ensure information provision concerning the virus and to help them continue their agricultural production as much as possible. Table 3 summarizes the practices the CSPs developed to build resilience.

**Table 3.** Overview of CSPs’ Practices of Resilience Building.

Forming unconventional alliances	Mobilizing digital technologies	Building subnetworks
CSPs partner with legal and psychosocial support organizations during the pandemic to help communities cope with the situation CSPs ally with religious leaders to inform communities about the virus and help them cope with it.	CSPs develop a radio broadcast based on Whatsapp to provide information about COVID-19 CSPs use other digital platforms to help farmers cope with the consequences of the pandemic and keep their agricultural production going.	CSPs create small communication networks to efficiently disseminate information about the virus throughout communities CSPs reorganize their training formats into smaller groups that continue their agricultural production.

### CSPs Develop New Capabilities During the Pandemic

#### CSPs Improve Their Ability to Include Stakeholders in Their Activities

As described above, the COVID-19 pandemic severely impacted the CSPs’ ability to continue their activities and compelled them to find ways to provide community support, nevertheless. While this was clearly challenging for them, several CSP members also described how this experience helped them to develop new skills, starting with an improved ability to involve their beneficiaries in the development of educational activities. For example, the radio show developed by the CSP in Uganda was so popular that its use was extended so that farmers could actively contribute to it. As the project coordinator explains:

We have continued Agrihour, but we have now even extended it with podcasts recorded by our local youth. Our goal is to produce about 30 episodes of 15 minutes. In these podcasts, we engage young people to talk about the central educational topics we cover in our programs, such as agriculture, gender balance, and water and health. The content is always adapted to the Agrihour show, so if the radio show is about clean water and sanitation, then the podcast will also cover this topic. (Project coordinator AgriU)

Besides enhancing their ability to involve farmers in the development of their activities, the CSPs also found new ways of connecting with external stakeholders. For instance, the CSP in Honduras found a new means of collaborating with external partners by using digital platforms. As the country manager indicates:

For us, something interesting that we saw with virtual training was that we have been able to have more partners involved in these sessions, because it was easier to coordinate with the government and with other institutions that were interested in giving more training. So, for example with some coffee institutes, we also started to develop online coffee diplomas and basically, what we saw was that we were able to involve many more partners than we had before. (Country manager AgriH)

#### CSPs Develop More Efficient Ways of Communicating With Beneficiaries

Aside from improving their ability to involve stakeholders in activities, the CSPs also discovered more efficient ways of communicating, especially with farmers. For one, the practice of restructuring communities into smaller networks has shown the CSPs the value of these structures in the long term. Below, the country manager of the CSP in Kenya explains:

What was helpful for us during the pandemic was building up strong networks of leaders within communities, the lead farmers, so to speak. This is a network we can use now in COVID-19 times but also moving forward, as it has shown us how much faster we can communicate. (Country manager AgriK)

Besides extending the use of small subnetworks and lead farmers, the use of digital technologies has enabled the CSPs to develop more efficient communication formats. For example, the country manager in Uganda recounts:

We saw how well things worked with phone farming and we noticed that many more young people were using WhatsApp than before, they created their groups, they were sharing things and so on. So, we organized a social media training within our community and now actually, they are also sharing more things with us than before the pandemic. (Country manager AgriU)

In conclusion, besides allowing them to remain functioning, the practices that the CSP engaged in to build resilience during the outbreak of COVID-19 have also allowed them to develop new capabilities, on which they can rely in the future.

## Discussion

### Resilience Building on a CSP Level

In this article, I set out to explore how CSPs that operate in a development context build resilience during times of adversity. Resilience is a topic that has received attention from the management literature on a variety of levels (Hillmann & Guenther, 2021; Linnenluecke, 2017; Raetze et al., 2021), yet studies examining such processes on a CSP level have been scarce. The findings of my study unfold three organizing practices that give rise to resilience on a cross-sectoral level: (1) forming unconventional alliances, (2) mobilizing digital technologies, and (3) building subnetworks.

A key question arising from these findings is what allowed the CSPs to engage in their practices of resilience building. Upon closer scrutiny, it emerges that the CSPs developed these by creatively combining physical resources and local knowledge. The example of “phone farming” illustrates the interplay of these two resources: once it became clear that the CSPs were no longer able to conduct field training with farmers because of access restrictions, local NGOs quickly mobilized business partners who ensured the provision of mobile devices. Yet, merely providing these devices would not have allowed for resilience building: based on their knowledge of farmers’ local circumstances and habits, NGOs and local CSP staff used these devices to develop a training format based on Whatsapp, which enabled the CSPs to largely continue their agricultural training with farmers. In a similar vein, for the practice of forming unconventional alliances, the CSPs’ business partners provided protective equipment, while local NGOs mobilized unconventional actors such as religious leaders, who were able to convince community members of the importance of using this equipment. Hence, by creatively combining physical resources provided by business partners with “non-physical local resources” (i.e., local values and knowledge (Shepherd & Williams, 2014), the CSPs were able to develop a set of practices that enabled them to remain functional amid the outbreak of the pandemic.

Extant studies on resilience organizing draw attention mainly to one specific resource or capability that allows organizations to build resilience, such as financial reserves (Bradley et al., 2011), adaptive business models (Hamel & Valikangas, 2003), or sensemaking capacities (Weick, 1993). As opposed to these studies, my findings show that for CSPs, it is not a single resource that gives rise to resilience, but its creative adaptation to their beneficiaries’ unique local context. In doing so, my findings underpin the importance of considering contextual factors to draw differentiated conclusions about how processes of resilience building unfold (Linnenluecke, 2017). In addition, whereas prior research has focused on the value of organizations’ relationships with internal stakeholders (e.g., employees) during crises (Gittell et al., 2006; Lengnick-Hall et al., 2011), my analysis shows that for CSPs, relationships with external stakeholders such as religious leaders or lawyers fulfill an important function in the process of resilience building. In a similar vein, the continuous involvement of community members played a key role in CSPs’ resilience building efforts, as the practices the CSPs developed were only effective to the extent that they were accepted and enacted by community members. All in all, my findings thus suggest that resilience building on a CSP level lies in the ability to creatively adapt physical resources to local circumstances, strengthening relations with external rather than internal stakeholders, and continuously involving beneficiaries in the process of resilience building.

Whereas combining physical resources with local knowledge enabled the CSPs to remain functional during the pandemic, they also managed to develop a set of new capabilities through this experience. Indeed, prior studies have emphasized how times of adversity can enable organizational growth, as such periods can serve as moments of “brutal audits” (Christianson et al., 2009, p. 850) that help organizational actors to re-evaluate their strengths and weaknesses. For the CSPs in this study, the need for resilience building enabled them to develop new skills, as it brought to the fore novel ways of interacting with farmer communities which were better aligned with the postpandemic environment. For instance, when the CSPs developed a radio broadcast to keep farmers and their families informed about the virus, they had not planned to continue this after the first weeks of the pandemic outbreak. Yet, the fact that a daily radio show proved to be an efficient educational format both for the CSPs and farmers, made the CSPs extend its use beyond the pandemic. In a similar vein, the fact that the younger generation of farmers became excited about using WhatsApp during the pandemic enabled the CSPs to develop new communication formats based on this technology. Thus, by detecting and seizing these new opportunities for interaction, the CSPs re-organized themselves in a more thorough and environmentally fitting way than before the pandemic (Lampel et al., 2009; Nava, 2022).

In addition to showing how CSPs can grow during periods of adversity, my findings bring clarity to the relationship between resilience (i.e., the ability to remain functional) on the one hand, and growth (i.e., the development of new capabilities) on the other. While these are often depicted as two distinct outcomes of resilience organizing (Hillmann & Guenther, 2021), the findings of this article suggest that resilience is an important condition that must be fulfilled for organizations to be able to develop new skills. Indeed, without a certain level of stability, the CSPs in this study would have not been able to identify future opportunities to engage with community members. Even though studies have suggested that resilience may reduce future learning opportunities (Nava, 2022), the findings of my study suggest the opposite, namely that a certain level of stability constitutes an important precondition for CSPs to grow during turbulent times.

**Figure 2.**
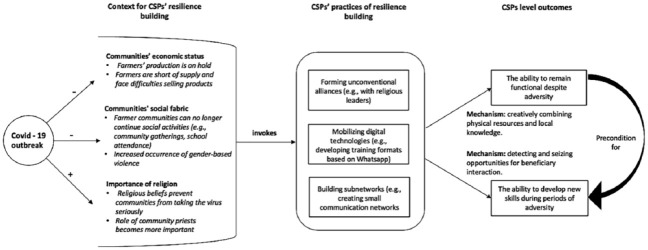
CSPs’ Resilience Building: Context, Practices, and Outcomes.

In sum, my findings unfold how the outbreak of the pandemic ensured that the CSPs found themselves in a context that required them to build resilience. In response, they developed three practices that enabled them to remain functioning and develop new skills during the pandemic. These outcomes were triggered by two different causal pathways: (a) creatively combining physical resources and local knowledge and (b) detecting and seizing novel opportunities for beneficiary interaction. Figure 2 provides an overview of these relationships.

### CSPs’ Incidental Learning

A final contribution offered by this study is the provision of a novel perspective on how CSPs engage in processes of collective learning. Scholars have pointed out that CSPs can act as important learning platforms for the actors involved (Dentoni et al., 2021) and have shown different ways of CSP learning. For one, studies have shown how partnerships collectively learn by engaging (external) actors such as boundary spanners, who liaise efficiently between the different partners involved (Ryan & O’Malley, 2016). Others have drawn attention to the importance of frequent experimentation to spur learning and change in CSPs (Berends et al., 2016; Dentoni et al., 2016). Finally, studies have highlighted how partners in CSPs learn collectively by deliberately reflecting on their own learning process and creating efficient routines for knowledge transmission, a process referred to as “triple loop learning” (Ameli & Kayes, 2011). While existing literature thus predominantly emphasizes the value of routinized and formalized efforts to cultivate CSP learning, the findings of my analysis sensitize us to the fact that learning may also occur unintentionally and as a side effect of other activities. For the CSPs in this study, this was clearly the case: by deviating from business as usual, these partnerships discovered novel ways of engaging with farmer communities, for example, by leveraging digital technologies or by restructuring communities’ communication networks. In addition, the experience during the pandemic made them discover more effective ways of beneficiary engagement.

As such, we can conceive of these unexpected outcomes as instances of incidental learning (i.e., learning outcomes that happen unintentionally and occur as a byproduct to other activities, Watkins & Marsick, 1992). By contrast to formalized or structured learning approaches, incidental learning occurs when actors are not aware of it and when learning is not the primary purpose of their activities (Drejer, 2000). To date, insights on incidental learning have mainly been developed in the context of workplace learning (Marsick et al., 2008) or education (Ledford et al., 2008), where scholars have shown how important learning outcomes can also be generated outside formally structured or classroom-defined activities. In addition, due to its unintentional nature, scholars have also argued that incidental learning can serve as an important means for actors to learn collectively under chaotic, or even hyper-turbulent conditions (Van Eijnatten & Putnik, 2004). Although more research is necessary to fully grasp the phenomenon of incidental learning in CSPs, the findings of my study provide a fruitful springboard to discuss alternative means of CSP learning, which has been emphasized as an important outcome of CSP collaboration (Austin & Seitanidi, 2012). My study shows how CSPs not only learn through formalized processes or trial and error experiences, but that learning can also happen unintentionally while CSPs undertake other activities.

## Limitations and Outlook

Like any empirical study, this article also has its limitations: first, it would have been valuable to include the beneficiaries’ perspective through primary data in my analysis. Even though I substituted for these missing perspectives by using other data sources, interviews with farmers would have provided valuable insights on the CSPs’ efforts of resilience building. Second, my analysis was restricted to a specific type of partnership operating in a development context. For future research, it would be valuable to assess how other CSP types, such as those that aim to develop sustainability standards for their members (De Bakker et al., 2019), or those focused on information sharing (Hahn & Pinkse, 2014), build resilience during turbulent times. In a similar vein, it is likely that other forms of organizing that involve collaboration with local communities might develop practices that are similar to those shown in this study. For example, firms that have a strong presence in communities (Muthuri et al., 2012; Valente, 2012) might also benefit from developing creative digital solutions to support these communities in times of adversity. Future research should thus assess how other forms of organizing that, like CSPs, closely interact with their beneficiaries build resilience during crises periods. Relatedly, as my analysis is restricted to the outbreak of the pandemic, I cannot draw conclusions on how this experience will have shaped organizing processes in the CSPs on a long-term basis. For future research, it would be valuable to explore resilience building in a longitudinal manner to assess whether and how CSPs organize for resilience during less turbulent times. Finally, I urge future research to explore the relevance and potential of digital technologies in a partnership context. Even though scholars have started to devote attention to this interplay (Rasche et al., 2021), it still provides ample opportunities for future research.

## Conclusion

In this study, I have explored how CSPs that provide support to local farmer communities organized for resilience during the COVID-19 pandemic. Drawing on eight CSPs operating in developing countries, I show how these partnerships developed three collaborative practices of resilience building: (a) forming unconventional alliances, (b) mobilizing digital tools, and (c) building subnetworks. Besides allowing the CSPs to remain functional despite the pandemic outbreak, the development of these practices enabled them to develop new capabilities for beneficiary engagement in the long term. This article offers two theoretical contributions: first, it widens our understanding of resilience building by showing how, on the level of CSPs, this process unfolds through a creative combination of physical resources and local knowledge. In addition, attention is drawn to the fact that CSPs can grow during periods of adversity by detecting and seizing opportunities for beneficiary interaction. Finally, this study shows that CSPs can learn unintentionally and as a byproduct of engaging in other activities, in this case, the attempt to continue functioning during a global pandemic. The main message of this article is that during periods of adversity, CSPs can remain functional if they manage to turn physical resources into creative, localized solutions. If, in this process, CSPs manage to detect and seize new opportunities for interacting with their beneficiaries, they may even emerge stronger from times of adversity such as a global pandemic.
